# Embedded ARM System for Volcano Monitoring in Remote Areas: Application to the Active Volcano on Deception Island (Antarctica)

**DOI:** 10.3390/s140100672

**Published:** 2014-01-02

**Authors:** Luis Miguel Peci, Manuel Berrocoso, Alberto Fernández-Ros, Alicia García, José Manuel Marrero, Ramón Ortiz

**Affiliations:** 1 Laboratory of Astronomy, Geodesy and Cartography, Department of Mathematics, Faculty of Science. Campus of Puerto Real, University of Cadiz, Puerto Real 11510, Spain; E-Mails: manuel.berrocoso@uca.es (M.B.); alberto.fernandez@uca.es (A.F.-R.); 2 Institute IGEO, CSIC-UCM. J. Gutierrez Abascal, 2, Madrid 28006, Spain; E-Mails: alicia.g@igeo.ucm-csic.es (A.G.); ramon.ortiz@csic.es (R.O.); 3 Volcanic Hazard and Risk Consultant, Los Realejos 38410, Spain; E-Mail: josemarllin@gmail.com

**Keywords:** ARM, multi-parameter system, volcanic activity, Linux Debian, environmental surveillance

## Abstract

This paper describes the development of a multi-parameter system for monitoring volcanic activity. The system permits the remote access and the connection of several modules in a network. An embedded ARM™™ processor has been used, allowing a great flexibility in hardware configuration. The use of a complete Linux solution (Debian™) as Operating System permits a quick, easy application development to control sensors and communications. This provides all the capabilities required and great stability with relatively low energy consumption. The cost of the components and applications development is low since they are widely used in different fields. Sensors and commercial modules have been combined with other self-developed modules. The Modular Volcano Monitoring System (MVMS) described has been deployed on the active Deception Island (Antarctica) volcano, within the Spanish Antarctic Program, and has proved successful for monitoring the volcano, with proven reliability and efficient operation under extreme conditions. In another context, *i.e.*, the recent volcanic activity on El Hierro Island (Canary Islands) in 2011, this technology has been used for the seismic equipment and GPS systems deployed, thus showing its efficiency in the monitoring of a volcanic crisis.

## 1. Introduction

Volcanoes are complex systems in which the diverse associated physico-chemical processes present a wide spatial variability [1]. In order to study these processes it is necessary to deploy monitoring networks, and the complexity of these networks will vary depending, *inter alia*, on the characteristics of the volcano and the perceived level of risk for the population [2]. In volcanic activity monitoring two objectives must be distinguished: research works and monitoring of the activity to forecast volcanic eruption and its communication to decision-makers. A number of different factors make the deployment of a network particularly complex; these include difficulties of physical access, extreme climate conditions, potential vandalism as well as data quality and remote data access in real time. Currently, there are multiple possibilities of data transmission in populated and developed areas. The design of a monitoring system depends heavily on the communication infrastructure available in the area (*i.e.*, in the Vesuvius volcano the monitoring network combines different technologies: analog Ultra High Frequency (UHF), digital UHF, Wireless Fidelity (Wi-Fi™) and Asymmetric Digital Subscriber Line (ADSL)) [3]. In unpopulated areas it is necessary to fully implement the communication system. When the topography permits, a ring of access points that surround the volcano is established. The seismic sensors with low power Wi-Fi™ links [4] and a Global Positioning System (GPS) network [5] are connected to this ring. With difficult topography, a detailed survey of the access points is needed [6]. The availability of different power Wi-Fi™ links has extended this technology to transfer data over a wide range of distances [7]. A recent development is the Optimized Autonomous Space *In-situ* Sensorweb (OASIS) system [8] showing possibilities of new technologies. It is designed with a dense array of sensors (distances between sensors of a few hundred meters) with short-range telemetry and low power consumption. Each node could communicate each other through a low-rate wireless area network. The topology used for data has been a dynamic tree of clusters rooted to a gateway. Data are collected using a light-weight adaptive linear predictive compression algorithm [9]. It is necessary to reach a compromise between data fidelity (quality and consistency of retrieved data) and yield (quantity of data delivered by the network) [10] together with the cost and energetic requirement of the system. In some cases, it is necessary to have an Air-dropped Sensor Network in order to properly instrument the volcanic area [11].

This paper presents a Multiparametric Volcanic Monitoring System (MVMS) for research and crisis management in any active volcanic area (dispersed volcanism, stratovolcanoes and/or volcanoes with persistent activity). The system is sufficiently flexible to quickly incorporate any sensor (seismometers, Global Navigation Satellite System-Global Positioning System (GNSS-GPS), inclinometers, infrasound, gas, Differential Optical Absorption Spectroscopy (MINIDOAS), *etc.*) and change the settings and applications adapting it to the available sensors and environmental conditions and activity. It also handles any type of link and stores the data locally to avoid losses during breaks in the links. It is capable of processing data in order to reduce the flow of data or automatically transmit warnings. It is low cost and requires low energy consumption. The MVMS has been specifically designed while conducting research projects (1995 to present) within the framework of the Spanish Antarctic Research Program, and has been applied to the active volcano of Deception Island (62° 77′S, 60° 37′W). The focus of these research projects is the monitoring and study of the volcanic activity, centered mainly on ground deformation [12–14] and seismicity [15,16]. The El Hierro Island (Canary Islands; 27.7° N; 18.0° W) unrest and eruption processes in 2011 [17–19] have allowed testing the effectiveness of this system for a rapid deployment of the monitoring network. In a first phase, the IESID module for measuring ground deformation was developed [20]. In a second phase, the parameters of seismic and weather information, ground temperature at different depths, heat flux and CO_2_ were added.

The use of different technologies in embedded systems has increased significantly in recent years; a great deal of development effort has been expended in a wide variety of applications, especially mobile phones, tablets, and cars; as a result, system costs have been considerably reduced. The wide range of possibilities now permits the scalability of a system, which must combine high processing and storage capability with low energy requirement. One of the main targets for development is the combination of embedded systems with communication (*i.e.*, Structural monitoring [21] and Earthquake Early Warning System [22]). In volcano monitoring [3–11] the main aim of the monitoring network is the detection in real-time of changes that may happen in the diverse parameters studied, *i.e.*, seismicity, ground deformation, temperature, gases, *etc.* [23]. In general terms, according to theoretical models of a volcanic system [24], before an eruption, activity increases very slowly at first, but shortly before the eruption, the rate of change in activity accelerates rapidly [25,26]. In order to detect the initial stages of unrest [27], it is necessary to have long time series of measurement data, which can be analyzed for very small changes with respect to the background level. The analysis of several parameters monitored simultaneously makes it possible to discern the volcanic origin of minor disturbances. It is also important to develop specific algorithms and to have sufficient capacity for the first data processing; thus the monitoring system can be automated to generate alerts that warn the team responsible about specific changes in the state of the volcano [18,28,29]. Other warnings are generated in case of the sensors or system malfunction [30]. Here, the main characteristics of the MVMS, particularly the acquisition, storage, processing and data transference, based on an embedded Advanced RISC Machine **(**ARM™) Atmel AT91SAM9G25 System-on-Chip (SoC) processor are described.

## 2. Modular Volcano Monitoring System (MVMS)

**Figure 1.**
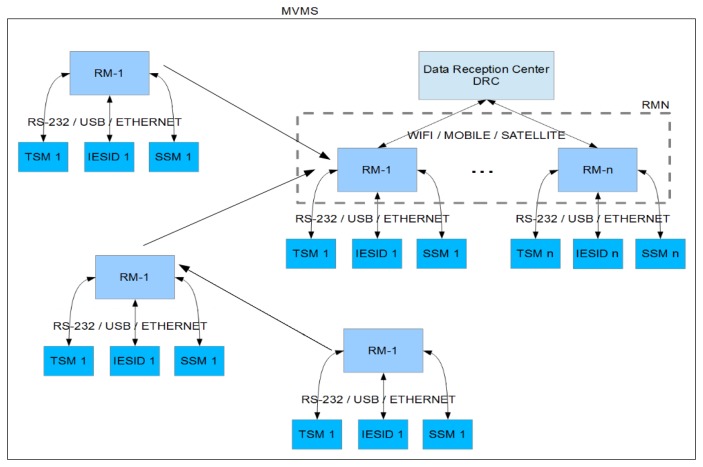
Components of the MVMS (block diagram of the modules).

The MVMS is composed of the Remote Modules Network (RMN) and the Data Reception Center (DRC). The RMN is formed by multiple modules for acquisition, storage and transmission of data from many diverse sensors. The DRC receives and processes the data and, in case of unrest or surveillance of an eruption, the Scientific Team (ST) analyzes in quasi real time the data and provides forecasts. Each module of the RMN (Remote Module, RM) includes an embedded ARM™ system and the communication system (see Figure 1). All these peripheral devices associated with the various different sensors are connected to this RM:

- Ground Deformation Module (IESID) described in [20];
- Thermometric Sensor Module (TSM);
- Seismic Sensor Module (SSM);
- Tide gauge;
- Other (webcam, magnetometer, self-potential, CO_2_, *etc.*).

The embedded ARM™ system has sufficient capacity to manage a seismic array, a powerful tool for the study of volcanic seismicity with cable connection [31–33] or Wi-Fi™ [34].

### 2.1. Hardware Components of the MVMS

The functions of RM are: sensor control, acquisition, storage and a first processing of data. In some cases data are sent periodically or under request of DRC. Furthermore, the DRC manages the warnings, which are automatically sent or triggers an alarm. For this reason, an embedded ARM™ system with enough storage and processing capacity has been selected. Depending on the needs of each location, the hardware characteristics of the embedded ARM™ system (processor speed, I/O ports, Universal Serial Bus (USB), Ethernet, video output, *etc.*) are chosen with the object of optimizing the power consumption/features. The same approach is adopted for the communication system: first the most appropriate type of link: Wi-Fi™, Bluetooth™, low-power Radio Frequency (RF), satellite, mobile phone, *etc.*, is chosen, and then the connection time and the link range are optimized, using different communication protocols (Security Shell (SSH), User Datagram Protocol (UDP), File Transfer Protocol (FTP), *etc.*). For this specific application, a Fox Board G25 from Acme Systems™ has been used [35]. G25 is a cost effective System-on-Module used to reduce the development time needed to design a low-power Linux embedded device. This system does not have a Graphic Processing Unit (GPU) that reduces power consumption. Power is supplied at 12V with a voltage range (see Table 1). G25 is available in two hardware configurations. The first one includes all peripherals (USB and Ethernet ports, Global System for Mobile Communications (GSM), GPS, temperature sensor, display, *etc.*) and it is the preferred option for development and evaluation of MVMS. The second option is set using the minimum necessary hardware and it has much lower power consumption. An Operating System (OS) for specific embedded ARM™ processors is used: EmDebian™ Grip Linux 7.1 “Wheezy” embedded device Linux Kernel version 3.11 [36,37]. This allows the configuration of the kernel to reduce the power consumption (also known as “low power modes”), adjusting the frequency and voltage of the processor. There are other embedded systems that use proprietary operating systems or limited versions of Linux [38–40]. In some cases, this represents a significant reduction in power; however, these systems are limited in on-board processing capabilities, storage and range wireless communications [34] so they cannot be used in our case.

All the RMs have been installed inside a robust and waterproof metal box for protection and insulation in extreme working conditions. As regards maintenance, in general, the RMs have I/O ports that can be configured as Analog/Digital Converters (ADC), sufficient for checking the state of the battery, system temperature, *etc.*, although they lack the precision and/or speed necessary for use with other types of sensor. Several different technologies are now available for the wireless transmission of data. These can be classified into three groups: Wi-Fi™, wireless modem and 3G mobile telephony. Mobile telephony only works in densely populated areas and, in the event of a natural or man-made disaster (earthquake, volcanic eruption, flood, explosion, *etc.*) it may be inoperative, as with Internet communication [23]. Wi-Fi™ technology is widespread, and a variety of devices with different functions is available. Radio modems use specific technologies so they must be carefully selected. These devices have low power consumption. Tables 2 and 4 summarize the main characteristics of two Wi-Fi™ modules and a radio modem tested.

**Figure 2.**
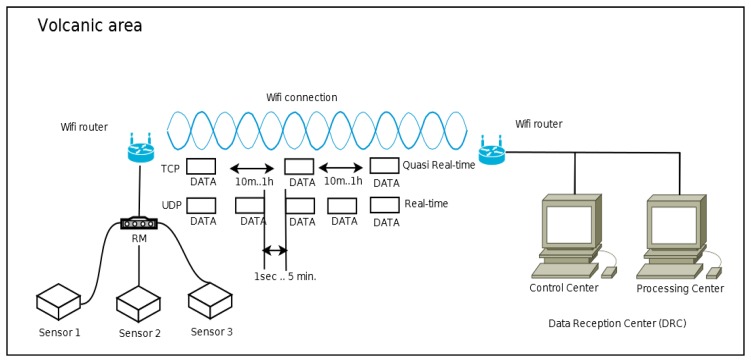
Real-time and quasi real-time operating modes.

**Figure 3.**
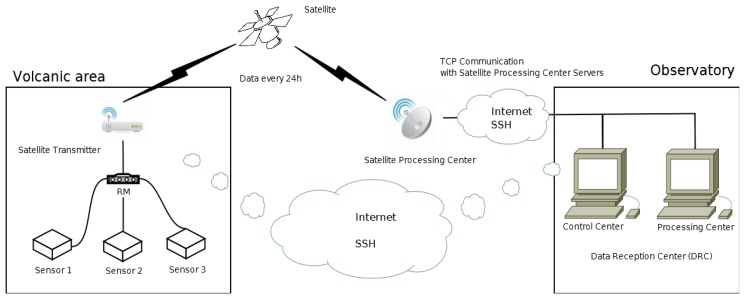
On demand operating mode.

The other main element of the MVMS is the DRC which manages and receives the data from RMs. Depending on the situation and the requirements there can be five different operating modes: real-time, quasi real-time, on-demand, maintenance and research mode. Each mode has the following characteristics:

1. Real-time operating mode. Direct connection to the sensors through the RM and re-sending of data in continuous mode using the UDP. The information sent is obtained from the data register for each sensor. The transmission of this data is very fast and needs low bandwidth but packet losses occur (see Figure 2);
2. Quasi real-time operating mode. Direct connection to the sensors through the RM and re-sending of data using the Transmission Control Protocol (TCP) with pre-set time intervals. The information sent is obtained from a file containing all the data for a period of either 10 min or one hour. This transmission handles a medium-size volume of data (see Figure 2);
3. On-demand operating mode. Direct connection to the sensors through the RM. Data requests are made using the TCP protocol, with longer time intervals which may or may not be programmed (on demand). The volume of the data sent corresponds to the time interval between requests, normally one day. A higher bandwidth and stable connection are needed (see Figure 3);
4. Maintenance operating mode. Includes maintenance required in the RM and in the data-logger of the sensors (e.g., deleting files, updating software, modifying settings, *etc.*). In general, a connection via SSH protocol is established, which permits the application to run in remote modes and/or sends files. Among the remote applications are the specific commands to access the settings of the sensors and check that everything is working correctly. Similarly each of the sensors can be restarted and it is possible to check the state of the general power supply, so that specific maintenance in the deployment area can be planned. The batteries will be changed when necessary;
5. Research operating mode. The system remains inactive for long periods of time, being activated only when the study area is visited. The data are stored in the RMs (off line) and they are downloaded manually. For scientific research only.

Depending on the data volume and/or level of volcanic activity, the reception and processing of the data can be centralized in a laptop or in a higher performance computer. In our specific case the DRC consists of a computer (INTEL™ i5) with the operating system based on Unix™ (in this case Ubuntu™ 12.04 LTS). This computer stores, processes and shares the data (e.g., Bernese 5.0™ for GPS data [41] and ObsPy [42,43], SEISAN™ [44] and Earthworm [45] for seismic data), and also manages the warnings [22]. In some case a computer network can be used in order to facilitate multiple users/accesses.

Equipment for use under extreme weather conditions must be designed with an autonomy that ensures safe operation during the pre-determined observation period. In the case of Antarctica, solar panels cannot be used in the winter, due to the absence of solar radiation. Wind turbines are not possible either, because ice accumulates on the blades. One of the solutions currently being tested is the exploitation of geothermal systems in volcanic areas to obtain power [46,47]. Each of the RM has its own power supply system, which protects the data independently of the RM. The power consumption of the ARM-module and the communication system is too high to operate for a whole year (more than 200 mAh). By reducing its working time to twenty minutes a day, (66.6 mAh), two batteries of 70 A/h in each RMN would provide enough power to work in winter. The data will be stored by the sensor data-loggers in its static First In First Out (FIFO) memory, so that these data will be ready to be sent when the ARM-module is activated. This operating method has been devised to reduce the power consumption of the MVMS to the minimum (see Table 5).

**Figure 4.**
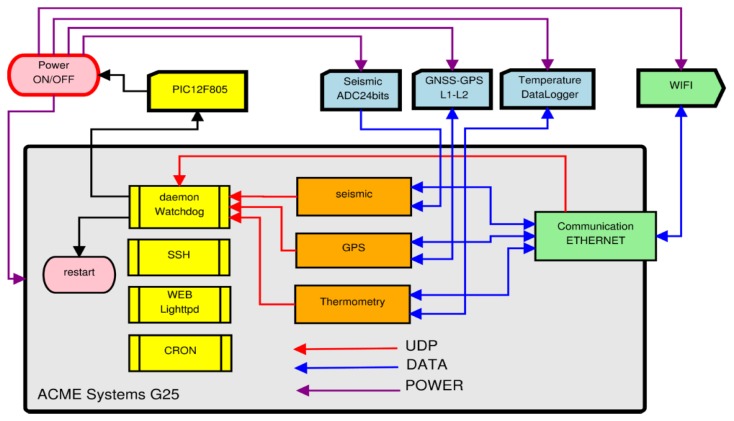
Watchdogs management.

A microcontroller (Microchip PIC12F508) is used as an external hardware watchdog system to reboot the system when the ARM-module hangs. In Figure 4, the hardware-software structure for the management of the watchdog is shown. All applications periodically send a UDP message to the watchdog daemon of the G25 ARM™ system. If the watchdog daemon receives all UDP messages in a time interval, it sends a pulse through a General-Purpose Interface (GPI) port that resets the watchdog external hardware (PIC12F508). Otherwise, the microcontroller cuts the power of the entire system (including sensors).

### 2.2. Software Components of the MVMS

At the software level, the MVMS consists of several applications distributed in the RMs and DRC system(s). In the Data Reception Center it is necessary to distinguish two types of applications:

- Basic Applications for receiving data, format changes and distribution to users.
- Applications processing and analysis.

The second group of applications is specific to the type of data and the needs of users and therefore is outside the scope of this paper. The RMs applications have been developed in Unix™ script, American National Standards Institute (ANSI) C [48], Python [49–51] and Java [52,53]. The Table 6 shows the main configure tasks for the ARM-module.

In general, the sensors used are commercial modules, which need the specific communication protocols. Seismic and GNSS-GPS systems have easy-to-integrate communication interfaces that use standard or well documented formats (Tables 7, 8 and 9).

In the case of thermometry, an application has been developed permitting the communication with the XR5 acquisition system of Pace Scientific™ [54] through its text command interface. The seismic instrumentation system has been designed within the CSIC-UCA research team using an inexpensive geophone SM6 4.5 Hz [55] with extended response to 0.5 Hz and a sigma-delta Analog-to-Digital Converter **(**ADC) (16/24 bits).

This instrumentation uses a very simple format (see Table 10) based on Earthworm to make data transfer easier and, in particular, to reduce the load in the microcontroller and to adjust its clock frequency in order to reduce the energy requirement.

The DRC must necessarily include the same software packages used for communication with RMs and libraries for decoding the data and messages received. The quantity and quality of the data depend on many factors starting from the sensor itself (*i.e.*, using a seismic sensor of 4.5 Hz or a Broadband, GPS L1 or L1L2), the number of samples to be stored and the number of bytes of each sample. This choice must combine data fidelity to the cost of the system and the energetic requirement, considering, at the same time, the loss of the device caused by volcanic activity or vandalism. Finally, both DRC and RMN incorporate a set of watchdog (software) that controls the proper operation of each of the components of the system, issuing warnings to ST in charge.

## 3. MVMS Applications: Deception Island (Antarctica) and El Hierro Island (Canary Islands)

**Figure 5.**
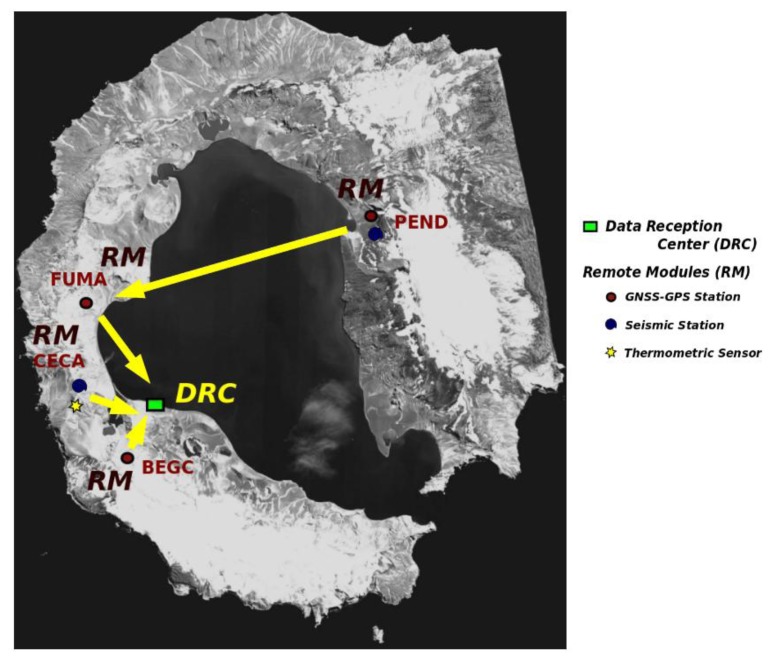
MVMS deployed on Deception Island. Distribution of RMs and DRC location. The GNSS-GPS stations form the IESID module; seismic stations form the SSM module and the thermometric sensors the TSM module.

This work has been carried out within the framework of volcanological research projects. Between other active volcanic areas, the MVMS has been developed and applied to study the geodynamic and volcanic activity of Deception Island (1995 to present, in the frame of Spanish Antarctic Research Program). More recently, this methodology has made possible a rapid instrumentation of El Hierro Island in 2011, during the unrest and eruption process. The MVMS deployed at Deception Island has been developed in successive phases [12–16] continually improving the hardware and software used. Figure 5 shows the configuration of the network in the 2012–2013 survey. Extended star topology is used. Distances from DRC to RMNs approximately of 10 km. The MVMS allows monitoring of both seismic activity and deformation in near real time.

**Figure 6.**
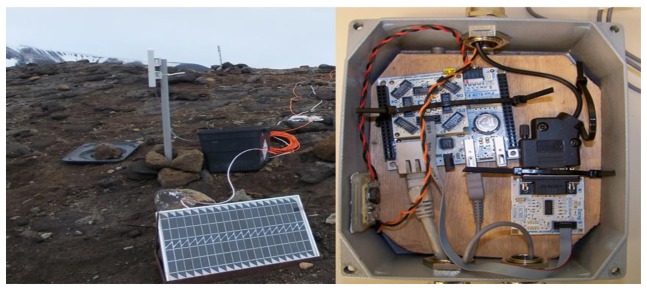
Detail of the transmission and power systems located in CECA and ARM™ module deployed.

The fast evaluation of volcanic activity on the island is a crucial factor for the safety of Antarctic researchers and, especially, the many tourists that visit the island every austral summer (3,286 in 2012–2013) [56]. Figure 6 shows the deployment of the GNSS-GPS station located in Cerro Caliente (CECA).

**Figure 7.**
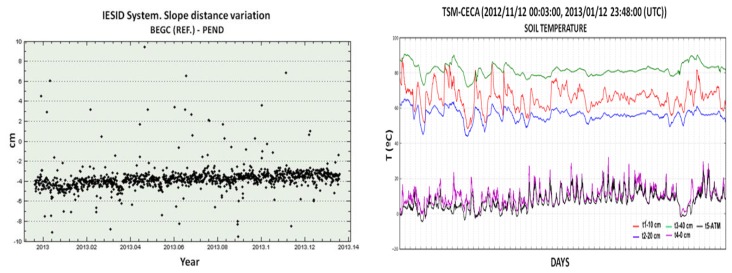
Records of the ground deformation parameter obtained by the MVMS system in the PEND station and thermometric anomalies in the CECA station.

At Deception Island the MVMS is powered by a 12 V 74 Ah battery for higher consumption components (ARM™, WI-FI™, and GPS-GNSS). For TSM and SSM sensors a 12 V and 7.2 Ah battery is used. A set of solar panels ensure the charge of the battery. The position data (GNSS-GPS data) are sampled at 1 Hz, the seismic data at 50 Hz and the temperature every 5 minutes. The sending of the GNSS-GPS data is carried out every hour even if the system supports the sending of position data every second. Seismic data are transmitted every second and temperature is transmitted every 5 min. Figure 7 shows an example of real time monitoring of ground deformation and temperature.

**Figure 8.**
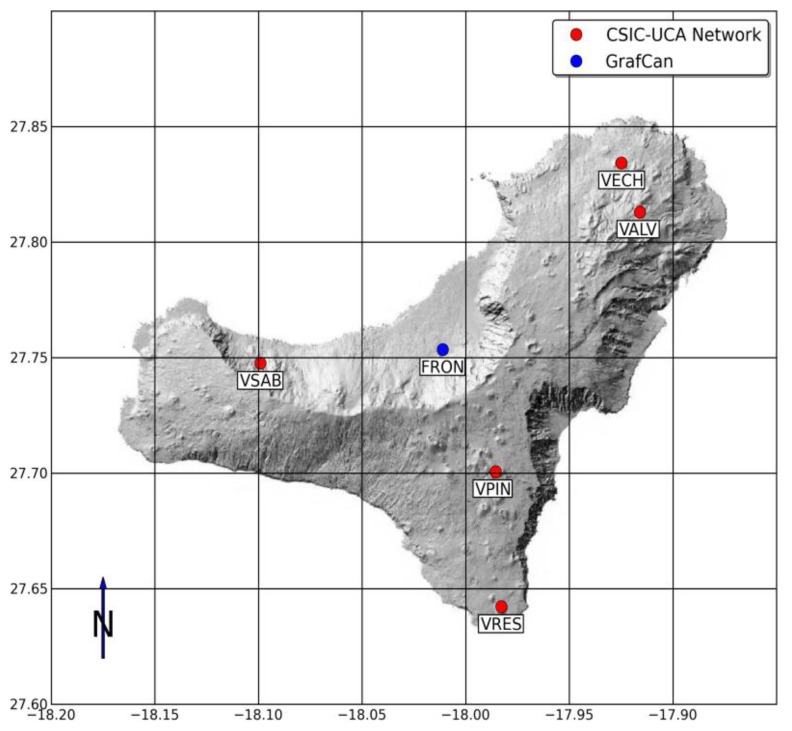
Distribution of the Remote Modules (CSIC-UCA network) on El Hierro Island. FRON is a GNSS-GPS public receiver (GRAFCAN, Canary Island Government). The complex topography of the island does not allow an easy implementation of a local network. Access to the data is carried out through Internet from the populated area nearest to RM.

In June–July 2011 period, an unrest process was detected on El Hierro Island [57] that demanded a quick instrument deployment that would allow near real-time monitoring of the activity. The instrumentation deployed was restricted to seismic sensors and GNSS-GPS (see Figure 8) with Internet access.

**Figure 9.**
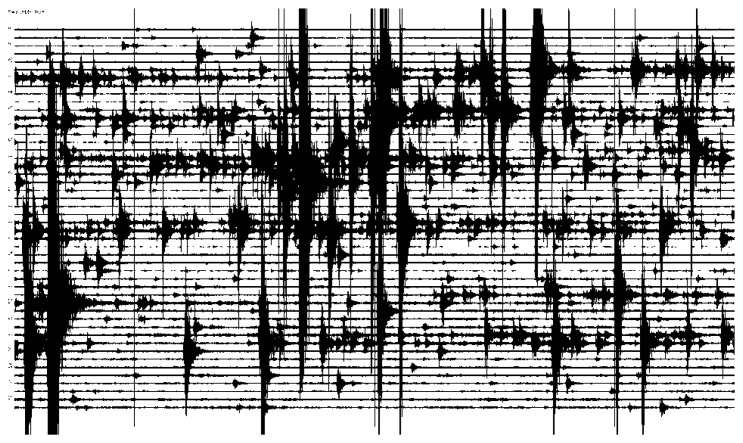
Seismic record of 2013/03/29, during the main magma injection process [18,19]. (VSAB station; CSIC-UCA network; El Hierro Island (See Figure 8)).

The seismic data transmission uses a UDP protocol every 5 s. Lost packets are recovered with SSH protocol. The GNSS-GPS data are accessed via FTP. Each RM is powered by a battery of 12 V and 12 Ah with a charger powered by the electric network ensuring the system operation during supply interruptions. The communications system and the ARM™ module operate in continuous mode to allow near real-time monitoring. In the moments of greatest activity Internet and mobile telephony were not available so the access to the stations was only possible with direct connection (wire or Wi-Fi™). Data from other stations required manual access (volunteers, citizen cooperation). Figure 9 shows a 24 h seismic record. This network allowed the scientific monitoring of the volcanic activity and the evolution forecast establishment [17–19].

## 4. Conclusions

This paper describes the development of an autonomous Monitoring Volcanic Multi-parameter System (MVMS) with remote access possibility and connection of several modules in a network. The MVMS is very easy to deploy for both research and/or monitoring network that uses the latest technologies of embedded systems, and to incorporate any type of sensor and/or communication system. Another advantage of MVMS is the possibility of a rapid development of the software necessary for managing the sensors and instruments available. The price of both hardware and software is very low. The MVMS consists of a network (or a network of networks) of Remote Modules (RMN) that receive the data via cable or through wireless links from sensors, store them locally on a large capacity support (Compact Flash (CF), Secure Digital (SD™), USB memory, *etc.*), make a first processing and send warnings. Data can be transmitted in near real time or on demand to a Data Reception Center (DRC). The local storage allows retrieving data when the transmission fails and uses only short transmission periods rather than continuous transmission. An embedded ARM™ system (highly reliable low-cost solution) with enough storage and processing capacity has been chosen. An Operating System (OS) for specific embedded ARM™ processors has been used: EmDebian™ Grip Linux 7.1 “Wheezy” embedded device Linux Kernel version 3.11 [36,37]. This OS offers great flexibility to develop applications related to different sensors (seismic, thermometry, GPS, *etc.*) including data processing and generation of warnings related to the change of activity in the volcanic system. It also allows the implementation of different communication protocols (satellite, telephony, Wi-Fi™, serial, *etc.*). ARM-modules can work as specific sensors networks (e.g., infrasounds, seismic array, *etc.*). Some ARM-modules can configure a network that sends data to a great capacity central module for data processing and transmission.

Because the MVMS is highly flexible, its deployment can be customized depending on the parameters that need to be measured for the particular phenomenon being studied for researching and/or crisis management. From the experience already gained, it can be stated that the deployment of embedded equipment based on ARM™ processors has proved to offer high reliability and moderate power consumption. The introduction of the new ARM™ processors and specific improvements in the Linux kernel for these microprocessors offer the prospect of greater energy efficiency. At the same time, battery technology is undergoing significant and rapid advances; these are developments that will make possible the unattended monitoring of modules of this type for more than a whole year, with a moderate cost, weight and volume in batteries.

The advantage of MVMS presented in this paper is that it allows the rapid development of a monitoring network that uses the latest technologies of embedded systems. These systems offer the possibility of developing the software necessary for managing the sensors and instruments available. The price of both hardware and software is very low.

The MVMS has been carried out within the framework of geodynamics and volcanic activity of Deception Island (Antarctica) and other research projects on active volcanic areas. Its most recent application has taken place during the 2011 unrest and eruption processes of El Hierro Island (Canary Islands) with very good results [17–19]. It is currently deployed on Tenerife Island to monitor the activity of the Teide volcano.

## Figures and Tables

**Table 1.** Characteristics of the Acmesystems™ G25 module.

**Component**	**Description**
Microprocessor	ATMEL™ ARM9 AT91SAM9G25 400 MHz
RAM memory	256 MB
MicroSD memory	16 GB
USB ports	×2 type 2.0; 12 MB
Ethernet ports	×1 10/100
Power requirement (no port activity)	80 mA

**Table 2.** Characteristics of the Ubiquiti™ NanoStation2 router.

**Characteristic**	**Value**
Range	15 km (with built-in antenna)
Antenna	Built-in 10 dBi + RP-SMA connector for external antenna
Max. power consumption	6 W
Power supply	12 V 1 A
Ethernet port	×1 10/100
Operating temperature	−40 to 85 °C
Operating humidity	5% to 95%
Interfaces	10/100 Base-TX (Cat. 5, RJ-45)
Operating frequencies	2,415 to 2,462 MHz
Sensitivity	RX −97 dBm ± 2 dBm
Power	TX 26 dBm, ± 2 dBm

**Table 3.** Characteristics of the TPLink™ TL-WA5210G router.

**Characteristic**	**Value**
Range	50 km (with built-in antenna)
Antenna	12 dBi
Max. power consumption	12 W (adjustable according to range)
Power supply	12 V 1 A
Ethernet port	1× RJ-45 10/100
Operating temperature	−30 to 70 °C
Operating humidity	10% to 90% no condensing
Interfaces	10/100 Base-TX (Cat. 5, RJ-45)
Operating frequencies	2.4 to 2.4835 GHz
Sensitivity	RX −98 dBm
Power	TX 27 dBm

**Table 4.** Characteristics of the YLX-TRM8053-500-05 and YLX-TRM8053-025-05 radio modems.

**Characteristic**	**YLX-TRM8053-500-05**	**YLX-TRM8053-025-05**
Range	40 km	2 km
Antenna	External	External
Max. power consumption	500 mW	25 mW
Power supply	2.4 to 3.6 V	2.4 to 3.6 V
Ethernet port	Na	Na
Operating temperature	Na	Na
Operating humidity	Na	Na
Interfaces	Through the pins of the chip	Through the pins of the chip
Operating frequencies	868 − 870/MHz	868 − 870/MHz
Sensitivity	−114 dBm	−114 dBm
Power	27 dBm	14 dBm

**Table 5.** Power requirement.

**RMN Subsystem**	**Current Requirement at 12 V**
GNSS -GPS System	* 430 mA
SSM Seismic System	6 mA
TSM Temperature System	30 μA
ARM Module Fox Board G25	*50–100 mA (depending on port activity)
WiFi Routers TPLink TL-WA5210G	* 1.0 A
Radio Modem YLX-TRM8053-500-05	* 0.5 A

Note:

* These modules can be activated for short periods of time in order to reduce the power consumption.

**Table 6.** ARM-module configuration.

**Linux Debian™ 7.1 “Wheezy”**	**Prebuilt Debian™ Packages Needed to Support Their Application**
Set system locale	en_US
Sensors	Set sensor folders tree, Specific sensor applications, I/O interfaces
Communication applications	IP address, Mode, Protocols, Security *etc.*
Set SSH	id_rsa.pub, ssh-keygen
Set deamons	Watchdog
Set crontab	Schedule commands to be executed periodically

**Table 7.** Earthworm format for seismic waveform.

**Field**	**Bytes**	**Type**
Pin Number	4	Integer
Number of samples in packet	4	Integer
Time of first sample in epoch seconds	8	Double
Time of last sample in epoch seconds	8	Double
Sample rate; nominal	8	Double
Site name	7	Char
Network name	9	Char
Component/channel code	9	Char
Data format code	3	Char
Data-quality field	2	Char
Padding	2	Char

**Table 8.** GPS format (Outside World Interface (OWI) Leyca Geosystems™). Sent by GPS GX1230 of Leica.

**Field**	**Size (bytes)**
Header	6/array of char
Id of message	3/array of char
Parameters	Variable number of parameters dependent of Id message
Checksum	4/integer

**Table 9.** Thermometric format sent by datalogger XR5.

**Field**	**Size (bytes)**
Date and time	19/array of char
Sensor1	8/float
Sensor2	8/float
Sensor3	8/float
Sensor4	8/float
Sensor5	8/float
Sensor6	4/integer
Sensor7	4/integer
Sensor8	8/float

**Table 10.** Format of the CSIC-UCA system.

**Field**	**Size (bytes)**
Header	4
GPS date and time	4
Delta of time	1
Station	4
Number of order	2
Bits	1
Channels	1
Data	50

## Acronyms

ARM: **A**dvanced **R**ISC **M**achine microprocessor architecture
DRC: **D**ata **R**eception **C**enter
GNSS-GPS: **G**lobal **N**avigation **S**atellite **S**ystem
IESID: **I**nclinometro **E**Spacial **I**sla **D**ecepcion (Deception Island Spatial Inclinometer)
MVMS: **M**odular **V**olcano **M**onitoring **S**ystem
RM: **R**emote **M**odule
RMN: **R**emote **M**odules **N**etwork
ST: **S**cientific **T**eam
SSH: **S**ecurity **S**hell
SSM: **S**eismic **S**ensor **M**odule
TSM: **T**hermometric **S**ensor **M**odule
CSIC-UCA: **C**onsejo **S**uperior **I**nvestigaciones **C**ientificas—**U**niversidad de **Ca**diz (Spanish Research Council – Cadiz University)

## References

[1] Seidl D., Hellweg M., Calvache M., Gomez D., Ortega A., Torres R., Böker F., Buttkus B., Faber E., Greinwald S. (2003). The multiparameter station at Galeras Volcano (Colombia): Concept and realization. J. Volcanol. Geotherm. Res..

[2] Ewert J., Guffanti M., Murray T. (2005). An Assessment of Volcanic Threat and Monitoring Capabilities in the United States: Framework for a National Volcano Early Warning System NVEWS.

[3] Orazi M., Peluso R., Caputo A., Capello M., Buonocunto C., Martini M., Marzocchi W., Zollo A. (2008). A Multiparametric Low Power Digitizer: Project and Results. Conception, Verification, and Application of Innovative Techniques to Study Active Volcanoes.

[4] Peluso R., Buonocunto C., Caputo A., De Cesare W., Orazi M., Scarpato G. (2009). Tecniche di Alta Disponibilità per l'acquisizione di dati sismici in ambiente GNU/Linux: Un'applicazione alla rete sismica di Stromboli. Quaderni di Geofisica.

[5] Puglisi G., Bonaccorso A., Mattia M., Aloisi M., Bonforte A., Campisi O., Cantarero M., Falzone G., Puglisi B., Rossi M. (2005). New integrated geodetic monitoring system at Stromboli volcano (Italy). Eng. Geol..

[6] Mattia M., Pellegrino D., Pulvirenti M., Rossi M. (2012). Applicazioni di sistemi di comunicazione wireless a 5 GHz per il monitoraggio multiparametrico dell'Etna.

[7] Scarpato G., de Cesare W., Orazi M., Peluso R., Caputo A., Martini M., Giudicepietro F. (2007). Sistemi di trasmissione WiFi per il monitoraggio sismico del Vesuvio.

[8] Song W., Hu X., Pan Y. Optimized Autonomous Space In-Situ Sensorweb, 2010.

[9] Huang R., Song W.Z., Xu M., Peterson N., Shirazi B., LaHusen R. (2012). Real-world sensor network for long-term volcano monitoring: Design and findings. IEEE Trans. Parallel Distrib. Syst..

[10] Werner-Allen G., Lorincz K., Johnson J., Lees J., Welsh M. Fidelity and Yield in a Volcano Monitoring Sensor Network.

[11] Song W., Huang R., Xu M., Ma A., Shirazi B., LaHusen R. ACM. Air-Dropped Sensor Network for Real-Time High-Fidelity Volcano Monitoring.

[12] Berrocoso M., Prates G., Fernández-Ros A., García A. (2012). Normal vector analysis from GNSS-GPS data applied to Deception Volcano surface deformation. Geophys. J. Int..

[13] Torrecillas C., Berrocoso M., Felpeto A., Torrecillas M., García A. (2012). Reconstructing palaeovolcanic geometries using a Geodynamic Regression Model (GRM): Application to Deception Island volcano (South Shetland Islands, Antarctica). Geomorphology.

[14] Prates G., Berrocoso M., Fernández-Ros A., García A. (2013). Enhancement of sub-daily positioning solutions for surface deformation monitoring at Deception volcano (South Shetland Islands, Antarctica). Bull. Volcanol..

[15] Vila J., Martí J., Ortiz R., García A., Correig A.M. (1992). Volcanic tremors at Deception Island (South Shetland Islands, Antarctica). J. Volcanol. Geotherm. Res..

[16] Ibáñez J.M., Del Pezzo E., Almendros J., La Rocca M., Alguacil G., Ortiz R., García A. (2000). Seismovolcanic signals at Deception Island volcano, Antarctica: Wave field analysis and source modeling. J. Geophys. Res..

[17] Prates G., García A., Fernández-Ros A., Marrero J.M., Ortiz R., Berrocoso M. (2013). Enhancement of sub-daily positioning solutions for surface deformation surveillance at El Hierro volcano (Canary Islands). Bull. Volcanol..

[18] Garcia A., Berrocoso M., Marrero J.M., Fernandez-Ros A., Prates G., De la Cruz-Reyna S., Ortiz R. (2013). Volcanic Alert System (VAS) developed during the (2011–2013) El Hierro (Canary Islands) volcanic process. Bull. Volcanol..

[19] Garcia A., Fernandez-Ros A., Marrero J.M., Berrocoso M., Prates G., De la Cruz-Reyna S., Ortiz R. (2011–2013). Magma displacements under insular volcanic fields, applications to eruption forecasting: El Hierro, Canary Islands. Geophys. J. Int..

[20] Peci L.M., Berrocoso M., Páez R., Fernández-Ros A., de Gil A. (2012). IESID: Automatic system for monitoring ground deformation on the Deception Island volcano (Antarctica). Comput. Geosci..

[21] Xu N., Rangwala S., Chintalapudi K.K., Ganesan D., Broad A., Govindan R., Estrin D. A Wireless Sensor Network for Structural Monitoring.

[22] Peng C., Zhu X., Yang J., Xue B., Chen Y. (2013). Development of an integrated onsite earthquake early warning system and test deployment in Zhaotong, China. Comput. Geosci..

[23] Bignami C., Bosi V., Costantini L., Cristiani C., Lavigne F., Thierry P. (2012). Handbook for Volcanic Risk Management—Prevention, Crisis Management, Resilience [Online].

[24] Voight B. (1998). A method for prediction of volcanic eruptions. Nature.

[25] De la Cruz-Reyna S., Reyes-Dávila G.A. (2001). A model to describe precursory material-failure phenomena: Applications to short-term forecasting at Colima volcano, Mexico. Bull. Volcanol..

[26] Ortiz R., Moreno H., García A., Fuentealba G., Astiz M., Peña P., Sánchez N., Tárraga M. (2003). Villarrica volcano (Chile): Characteristics of the volcanic tremor and forecasting of small explosions by means of a material failure method. J. Volcanol. Geotherm. Res..

[27] Tárraga M., Carniel R., Ortiz R., García A. (2008). The failure forecast method: Review and application for the real-time detection of precursory patterns at reawakening volcanoes. Dev. Volcanol..

[28] Fearnley C. (2011). Standardising the USGS Volcano Alert Level System: Acting in the Context of Risk, Uncertainty and Complexity. Ph.D. Dissertation.

[29] Fearnley C., McGuire W., Davies G., Twigg J. (2012). Standardisation of the USGS Volcano Alert Level System (VALS): Analysis and ramifications. Bull. Volcanol..

[30] Peng Y., Lahusen R., Shirazi B., Song W. IET. Design of Smart Sensing Component for Volcano Monitoring.

[31] Ortiz R., García A., Olmedillas J.C., Vila J. (1991). Portable digital seismic array for volcano monitoring. Les Cahiers du Centre Europeen de Geodinamique et Seismologie.

[32] Almendros J., Ibañez J., Alguacil G., Del Pezzo E., Ortiz R. (1997). Array tracking of volcano tremor source at Deception Island, Antarctica. Geophys. Res. Lett..

[33] Del Pezzo E., La Rocca M., Petrosino S., Grozea B., Maritato L., Saccorotti G., Simini M., Ibañez J., Alguacil G., Carmona E. (1998). Twin Digital Short Period Seismic Array Experiment at Stromboli Volcano.

[34] Nittel S. (2009). A survey of geosensor networks: advances in dynamic environmental monitoring. Sensors.

[35] Acme Systems SRL. http://www.acmesystems.it/.

[36] Debian Operating System. http://www.debian.org/.

[37] Embedded Debian Project. http://www.emdebian.org/.

[38] TinyOS Open Source Operating System. http://www.tinyos.net/.

[39] Lantronix, Inc.. http://www.lantronix.com/.

[40] Contiki Open Source Operating System. http://www.sics.se/contiki.

[41] Dach R., Hugentobler U., Fridez P., Meindl M. (2007). Bernese GNSS Software, Version 5.0.

[42] Beyreuther M., Barsch R., Krischer L., Megies T., Behr Y., Wassermann J. (2010). ObsPy: A python toolbox for seismology. Seismol. Res. Lett..

[43] Megies T., Beyreuther M., Barsch R., Krischer L., Wassermann J. (2011). ObsPy—What can it do for data centers and observatories?. Ann. Geophys..

[44] Ottemöller L., Voss P., Havskov J. (2013). Seisan Earthquake Analysis Software for Windows, Solaris, Linux and Macosx [Online].

[45] Johnson C.E., Bittenbinder A., Bogaert B., Dietz L., Kohler W. (1995). Earthworm: A flexible approach to seismic network processing. Iris Newsl..

[46] Bhushan B. (2010). Springer Handbook of Nanotechnology.

[47] Sveinsson J., Gudmundsson M.T., Palsson F. A Geothermally Driven Peltier Generator for Powering Instruments and Transmission Link from Vantajokull Glacier (Veggspjald).

[48] Kernighan B.W., Ritchie D.M. (1988). The ANSI C Programming Language.

[49] Jones E., Oliphant T., Peterson P. (2001). SciPy: Open Source Scientific Tools for Python.

[50] McKinney W. (2012). Python for Data Analysis.

[51] Hunter J.D. (2007). Matplotlib: A 2D graphics environment. Comput. Sci. Eng..

[52] Flanagan D. (2009). Java Examples in a Nutshell.

[53] Gosling J., Joy B., Steele G., Bracha G., Buckley A. (2013). The Java Language Specification, Java SE.

[54] Pace Scientific Data Loggers and Sensors. http://www.pace-sci.com/.

[55] SM-6 Geophone. http://www.iongeo.com/content/includes/pdfs/SM6_121026.pdf.

[56] International Association of Antarctica Tour Operators. http://iaato.org/es/tourism-statistics.

[57] López C., Blanco M.J., Abella R., Brenes B., Cabrera V.M., Casas B., Domínguez I., Felpeto A., Fernández M., del Fresno C. (2012). Monitoring the volcanic unrest of El Hierro (Canary Islands) before the onset of the 2011–2012 submarine eruption. Geophys. Res. Lett..

